# Effect of morbidity on the association between lifestyle and mortality in the Health Examinee cohort

**DOI:** 10.4178/epih.e2026022

**Published:** 2026-05-27

**Authors:** Abraham Kaggwa, Anthony Kityo, Sang-Ah Lee

**Affiliations:** 1 Interdisciplinary Graduate Program in Medical Bigdata Convergence, Kangwon National University College of Medicine, Chuncheon, Korea; 2 Department of Preventive Medicine, Kangwon National University College of Medicine, Chuncheon, Korea

**Keywords:** Multimorbidity, Life style, Mortality, Life expectancy, Cohort studies

## Abstract

**OBJECTIVES:**

Healthy lifestyles are associated with lower mortality risk, but evidence among Asian populations with multimorbidity remains limited. We examined the association between lifestyle and all-cause mortality, stratified by morbidity status.

**METHODS:**

We analysed 111,537 Korean adults aged 40–69 years from the Health Examinee cohort (2004–2020). Five lifestyle factors—smoking (pack-years), alcohol intake (g/day), physical activity (MET-hr/day), sleep duration (hr/day), and diet quality, assessed using the Korean Diet Quality Index—were standardised and regressed on mortality. Regression coefficients were positive for high-risk behaviours and negative for protective behaviours; these coefficients were summed to generate a weighted lifestyle score (WLS), with higher values indicating a less healthy lifestyle. We estimated hazard ratios (HRs) per standard deviation (SD) increase in WLS and across WLS tertiles (T1=healthiest, T3=least healthy), as well as years of life lost (YLL), stratified by morbidity status (none, 1 condition, or multimorbidity).

**RESULTS:**

During a median follow-up of 10.6 years, 3,366 deaths occurred. A higher WLS was associated with a 21% increase in mortality risk among participants with multimorbidity. HRs and 95% confidence intervals (CIs) for T3 versus T1 were 1.40 (95% CI, 1.13 to 1.74) among participants with no disease, 1.68 (95% CI, 1.39 to 2.03) among those with 1 disease, and 1.62 (95% CI, 1.31 to 2.01) among those with multimorbidity. Associations were stronger among females with multimorbidity. At age 40 years, T3 was associated with YLL of 4.9 years in males and 4.3 years in females with multimorbidity.

**CONCLUSIONS:**

A higher lifestyle score, indicating a less healthy lifestyle, was associated with reduced life expectancy, particularly among individuals with multimorbidity. These findings highlight the potential benefits of adopting a healthy lifestyle in this population subgroup.

## GRAPHICAL ABSTRACT

[Fig f5-epih-48-e2026022]

## Key Message

The prevalence of multimorbidity is rising rapidly in Korea, yet evidence on the impact of lifestyle on life expectancy remains limited. Using a weighted lifestyle score, this study shows that unhealthy behaviours are associated with increased mortality across all morbidity levels and with shortened life expectancy of up to 5 years, particularly among individuals with multimorbidity. These findings support the relevance of lifestyle behaviours in populations with chronic disease.

## INTRODUCTION

Modifiable lifestyle factors, including smoking, poor diet, physical inactivity, alcohol use, and suboptimal sleep duration, are major determinants of chronic disease and important prognostic markers of mortality [1]. Although these behaviours independently influence risk, they often cluster in real-world settings [2], resulting in synergistic effects on disease risk. Adoption of multiple low-risk behaviours has been associated with substantial gains in life expectancy. For example, adopting at least 4 low-risk behaviours—non-smoking, a healthy diet, regular physical activity, and moderate or no alcohol consumption—has been shown to reduce all-cause mortality risk by 66% [3].

Most previous studies of healthy lifestyles have focused on generally healthy individuals [1,4-11], limiting their applicability to individuals with multimorbidity. With increasing global life expectancy [12], the population profile has shifted towards a greater burden of multimorbidity, defined as the coexistence of 2 or more chronic conditions [13,14]. Multimorbidity affects approximately 37.2% of the global population [15] and up to 40% of the Korean population [16]. In this population, lifestyle factors do not merely coexist with disease but may act as shared biological drivers of systemic inflammation and metabolic dysfunction [17]. These behaviours may accelerate the pathophysiological progression of existing conditions, thereby compounding the baseline mortality risk associated with multimorbidity [18].

Despite this, evidence remains limited on how combined lifestyle patterns mitigate risk in multimorbid Asian populations, including Korea, which is undergoing a rapid demographic transition [19]. Most conventional indices assign equal weights to each behaviour through simple summation [20]. This approach is limited because it assumes that each behaviour has an equivalent biological impact on prognosis. In reality, dominant factors such as smoking exert a greater influence on mortality than other factors, such as sleep duration [21]. Therefore, simple summation may dilute the true prognostic signal and reduce the precision of risk stratification. Furthermore, although sleep duration is increasingly recognised as an important determinant of mortality [22], it has been omitted from many composite indices [10,20].

Therefore, refined weighted lifestyle scores (WLSs) that integrate sleep and account for the differential contribution of each factor are needed to provide more meaningful risk stratification. We tested the hypothesis that a WLS—a standardised continuous measure in which higher values indicate a less healthy lifestyle profile and that combines smoking, alcohol intake, diet quality, physical activity, and sleep duration—is significantly associated with increased all-cause mortality across different morbidity levels. Our objectives were to: (1) compute a standardised WLS; (2) examine its association with all-cause mortality, stratified by morbidity status; and (3) quantify the years of life lost (YLL) associated with a higher, less healthy WLS using a large-scale Korean cohort.

## MATERIALS AND METHODS

### Study design and population

We used the Health Examinee (HEXA) cohort, a subcohort of the Korean Genome and Epidemiology Study (KoGES), which was designed to examine lifestyle and genetic risk factors for chronic disease in Korea [23]. Between 2004 and 2013, 173,195 individuals aged 40–69 years were recruited from 38 health centres and hospitals across Korea; of these, 130,219 consented to death-record linkage [24].

**Figure 1.**
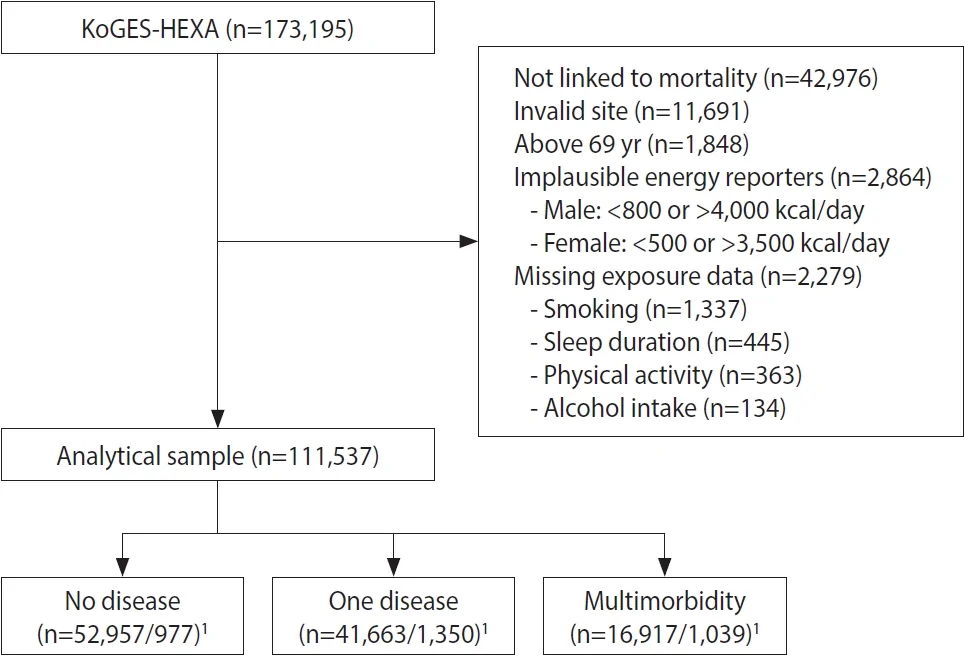
Flow diagram of sample selection criteria. KoGES-HEXA, Korean Genome and Epidemiology Study- Health Examinee. 1Number of events.

We excluded 11,691 participants from invalid recruitment sites, defined as pilot sites that did not meet biospecimen quality-control standards or had less than 2 years of participation [25]. We also excluded 1,848 participants outside the 40–69-year age range, 2,864 with implausible energy intake (<800 or ≥4,000 kcal/day for males; <500 or ≥3,500 kcal/day for females) [26], and 2,279 with missing lifestyle data on smoking, drinking, sleep duration, or exercise. The final analytical sample comprised 111,537 participants, of whom 52,957 (47.5%) had no chronic disease, 41,663 (37.4%) had 1 disease, and 16,917 (15.2%) were classified as having multimorbidity (≥2 diseases) (Figure 1).

### Ascertainment of mortality

Deaths occurring between 2004 and 2020 were identified using unique identifiers through linkage to the Korean National Statistical Office death registry or the Korean National Health Insurance Corporation.

### Assessment of lifestyle factors

Baseline lifestyle data were collected using a standardised interviewer-administered questionnaire [24,27,28]. Smoking status was categorised as never, former, or current smoking, and cumulative exposure was expressed as pack-years using smoking frequency, amount, and duration [24]. Alcohol consumption status was categorised as current, former, or never drinking [27]. For current drinkers, daily intake (g/day) was estimated from drinking frequency, volume, and beverage-specific alcohol content [29].

Physical activity was measured using the Minnesota Leisure-Time Physical Activity questionnaire [24], expressed in MET-hr/day [28] and classified as active (≥3 MET-hr/day) or inactive (<3 MET-hr/day) [30]. Sleep duration was self-reported and categorised as short (<7 hours), normal (7–8 hours), or long (>8 hours) [31]. Dietary quality was assessed using a validated 106-item semi-quantitative food-frequency questionnaire [32] and summarised using the Diet Quality Index for Koreans (DQI-K; range, 0–9); scores >5 indicated good diet quality [33].

#### Construction of lifestyle score

We developed 2 composite indices to characterise baseline lifestyle. For the primary analysis, a WLS was derived by weighting each lifestyle factor, treated as a continuous variable, according to its independent association with all-cause mortality. The 5 lifestyle factors were standardised and jointly entered into a Cox regression model with restricted cubic splines [21]. The resulting regression coefficients (β) served as weights: positive coefficients were assigned to high-risk behaviours (smoking, alcohol use, and suboptimal sleep), and negative coefficients were assigned to protective behaviours (physical activity and diet quality). The WLS was calculated by summing the weighted standardised lifestyle variables, with higher scores indicating a less healthy lifestyle [34]. Model specifications and coefficients are provided in Supplementary Material 1.

For comparability with traditional studies, the accumulated lifestyle score (ALS) was computed as a simple count of unhealthy behaviours (0–5) [35]. Each factor was dichotomised into high-risk (1) versus low-risk (0). High-risk behaviours were defined as current or former smoking, current drinking, physical activity <3 MET-hr/day, suboptimal sleep (<7 or >8 hours), and poor diet quality (DQI-K ≤5) [35]. A summary diagram of the ALS derivation procedure is shown in Supplementary Material 2. For analysis, ALS was categorised as ≤2 (reference), 3, or 4–5 to optimise the sample size within categories.

### Assessment of multimorbidity

Chronic morbidity status was assessed at baseline using self-reported physician diagnoses and medication use [24]. Conditions included hypertension, diabetes, dyslipidaemia, cardiovascular disease (myocardial infarction and ischaemic stroke), cancer, respiratory disease (asthma, bronchitis, and chronic pulmonary disease), gastrointestinal disorders (ulcer and gastritis), thyroid disease, gout, gallstones, fatty liver disease, chronic kidney disease, prostate disease, cystitis, arthritis, osteoporosis, glaucoma, and Parkinson’s disease.

Objective measurements were also applied where available. Hypertension was defined as systolic blood pressure ≥130 mmHg, diastolic blood pressure ≥85 mmHg, or use of antihypertensive medication. Diabetes was defined as fasting glucose ≥126 mg/dL. Chronic kidney disease was defined as an estimated glomerular filtration rate (eGFR) <60 mL/min/1.73 m^2^, estimated using the Chronic Kidney Disease Epidemiology Collaboration equation [36].

A morbidity count was calculated by summing conditions (presence=1, absence=0) and categorised as 0 (no chronic disease), 1 (one disease), or ≥2 (multimorbidity). Multimorbidity was defined as the coexistence of 2 or more of these 18 chronic conditions [13,14].

### Statistical analysis

Participants were followed from baseline until death or December 31, 2020, whichever occurred first. Follow-up time was calculated as the difference between age at recruitment and age at the end of follow-up. Baseline characteristics were summarised as counts and percentages for categorical variables and as medians with interquartile ranges (IQRs) for continuous variables. Missing covariates (body mass index [BMI], 0.03%; waist–hip ratio [WHR], 0.24%; marital status, 0.24%; education, 0.75%; income, 8.30%) were imputed using the mode within sex-specific and age-specific strata. Participants were classified into WLS tertiles (T1–T3), with higher tertiles indicating a less healthy lifestyle.

Associations between lifestyle scores and all-cause mortality were estimated using multivariable Cox proportional hazards models, with age as the timescale, stratified by morbidity status (none, 1 condition, or multimorbidity) and sex. For the WLS, HRs were estimated per SD increase and by tertile (T2 and T3 vs. T1). For the ALS, HRs were estimated for categories 3 and 4–5 versus ≤2. Models were adjusted for sex (except in sex-specific models), region of residence, education level, marital status, household income, BMI, WHR, and total energy intake.

Life expectancy was estimated using flexible parametric survival models with age as the timescale [21]. YLL were calculated as the difference in remaining life expectancy between the less healthy groups (WLS: T2/T3; ALS: 3/4–5) and the healthiest reference groups (WLS: T1; ALS: ≤2).

Sensitivity analyses were conducted by excluding deaths that occurred within the first 2 years of follow-up to account for sick-quitter bias and potential reverse causation.

All Cox regression models were built using SAS version 9.4 (SAS Institute Inc., Cary, NC, USA), and flexible parametric survival models were fitted in R version 4.3.0 (R Foundation for Statistical Computing, Vienna, Austria) using the rstpm2 package.

### Ethics statement

The study was approved by the Institutional Review Board of Kangwon National University Hospital (KWNU IRB-2022-07-007). The KoGES cohort was approved by the Korean National Institute of Health and the institutional review boards of all participating hospitals (IRB No. E-1503-103-657). Written informed consent was obtained from all participants before enrolment.

## RESULTS

### Baseline characteristics

The median age of participants was 54 years (IQR, 49–59) in males and 52 years (IQR, 47–57) in females. Participants in the healthiest tertile (T1) were older, more highly educated, more likely to be married, and had higher incomes than those in the least healthy tertile (T3). In males, less healthy lifestyles were associated with lower educational attainment, higher BMI, higher WHR, and a greater prevalence of multimorbidity. In females, central obesity was common across all tertiles, whereas multimorbidity was less frequent in T3 than in T1 (Table 1).

Hypertension was the most common chronic condition, affecting 76% of participants with at least 1 disease (81% of males and 74% of females). Diabetes (5%), chronic kidney disease (3%), arthritis (2%), and thyroid disease (2%) were the next most common conditions. Disease prevalence varied by sex: osteoporosis, arthritis, and thyroid disease were more common in females, whereas diabetes, myocardial infarction, and prostate hypertrophy were more common in males. Among participants with multimorbidity, frequent clusters included hypertension with diabetes (20%), hyperlipidaemia (10%), and chronic kidney disease (10%) (Supplementary Material 3). During a median follow-up of 10.6 years (IQR, 9.3–11.9; 1.18 million person-years), 3,366 deaths occurred. Of these, 977 occurred among participants without chronic disease (522 in males and 455 in females), 1,350 among those with 1 disease (825 in males and 525 in females), and 1,039 among those with multimorbidity (659 in males and 380 in females).

### Weighted lifestyle score and mortality by morbidity status

**Figure 2.**
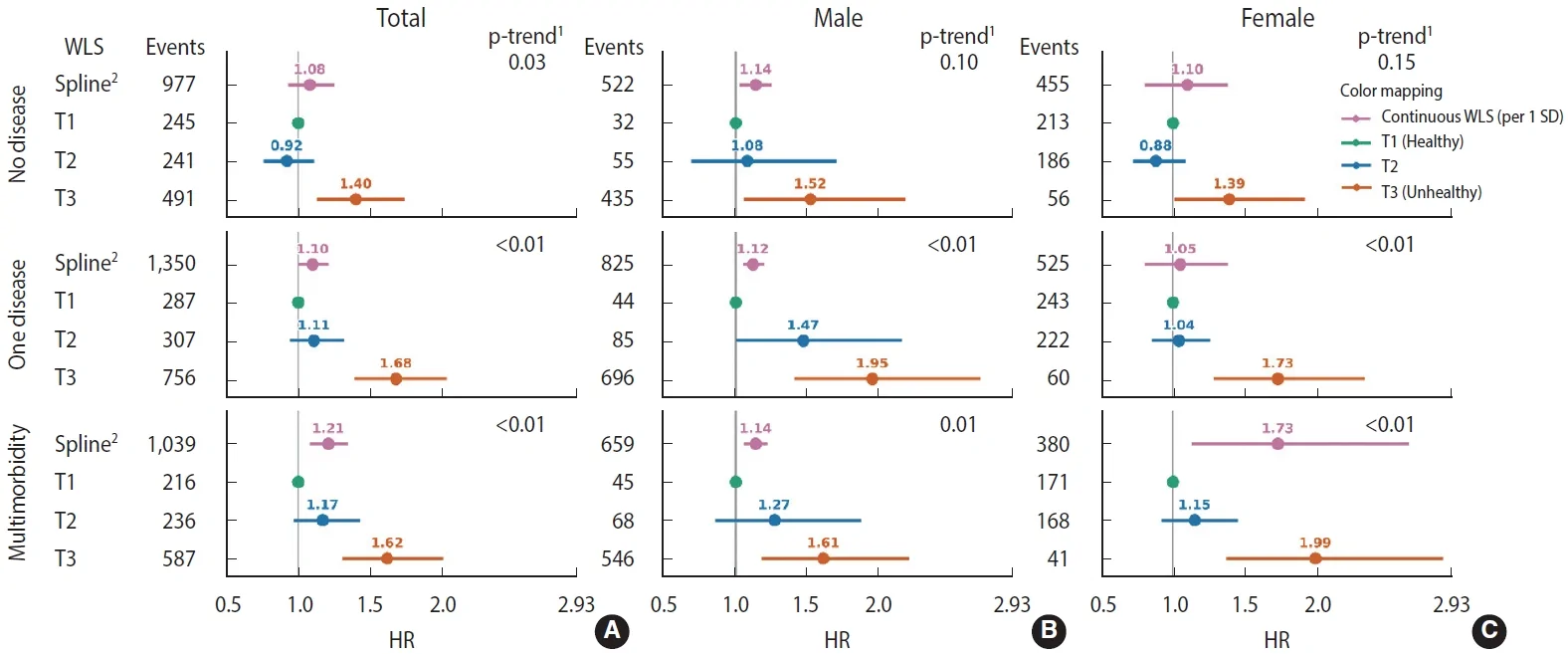
HRs for mortality by WLS according to morbidity status (A: total, B: male, C: female). The WLS was developed to assess the varying impact of lifestyle factors on mortality risk. Each lifestyle factor was standardised as a z-score and weighted using regression coefficients derived from cubic spline models adjusted for sex, BMI, WHR, energy intake, morbidity status, education, income, marital status, and region. The weighted scores were then summed to calculate the WLS. HRs and 95% CIs were adjusted for energy intake, sex (in both-sex combined models), education, region of residence, marital status, income, BMI, and WHR. Tertile 1 indicates the healthiest lifestyle, and tertile 3 indicates the least healthy lifestyle. HR, hazard ratio; CI, confidence interval; SD, standard deviation; WLS, weighted lifestyle score; BMI, body mass index; WHR, waist–hip ratio. ^1^p-values were derived from models treating WLS as an ordinal variable to assess linear trends across tertiles. ^2^Restricted cubic spline transformation used to estimate the regression coefficients for each continuous lifestyle factor in the multivariable Cox proportional hazards model.

In the primary analysis using the WLS, a higher WLS, indicating a less healthy lifestyle, was associated with higher mortality across morbidity groups and in both males and females (Figure 2, Supplementary Material 4). A 1-SD increase in WLS was associated with a 12–14% increase in mortality risk among males and a 73% increase among females with multimorbidity. Categorical analysis confirmed this trend: compared with the healthiest tertile (T1), the least healthy tertile (T3) was associated with significantly higher mortality risk in males with multimorbidity (HR, 1.61; 95% CI, 1.18 to 2.21) and females with multimorbidity (HR, 1.99; 95% CI, 1.37 to 2.88). Significant linear trends (p for trend <0.01) were observed in all groups with existing morbidity, whereas the association was less pronounced in the healthiest strata (p for trend >0.05).

### Accumulated lifestyle score and mortality by morbidity status

**Figure 3.**
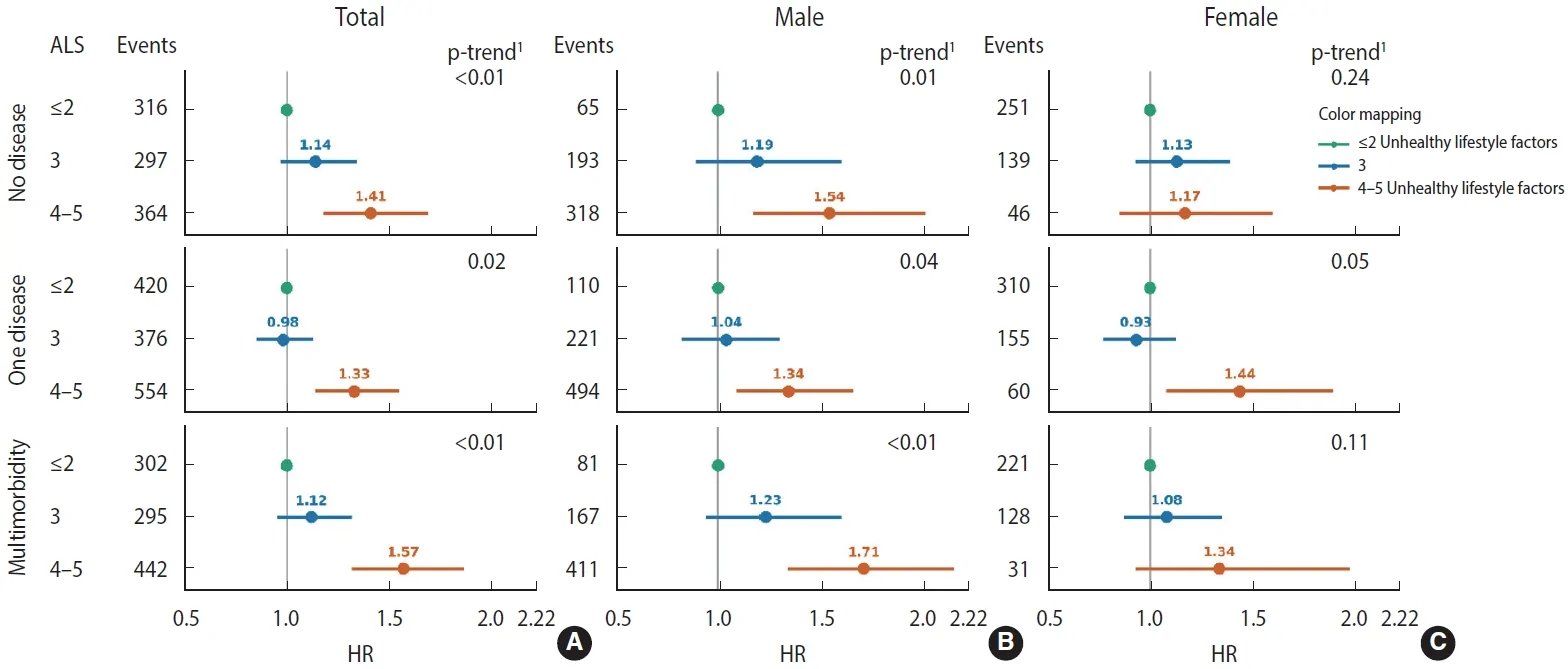
HRs for mortality by accumulated lifestyle score according to morbidity status (A: total, B: male, C: female). HRs were adjusted for energy intake, sex (in both-sex combined models), education, region of residence, marital status, income level, BMI, and WHR. The ALS ranges from 0 to 5 and was calculated by summing binary exposures (0/1) for the following behaviours: smoking, alcohol use (current drinking), poor diet quality (DQI-K ≤5), low physical activity (MET-hr/day <3), and short or long sleep duration. Participants received 1 point for each risk behaviour present. HR, hazard ratio; ALS, accumulated lifestyle score; BMI, body mass index; WHR, waist–hip ratio. ^1^p-values represent the p for linear trend across increasing numbers of unhealthy lifestyle factors, grouped as ≤2, 3, and 4–5.

To provide context relative to traditional count-based indices, we analysed the ALS (Figure 3, Supplementary Material 5). Although directionally similar to the WLS, the ALS showed different patterns of statistical significance across morbidity groups. For example, in females with multimorbidity, the ALS association did not reach statistical significance (HR, 1.34; 95% CI, 0.93 to 1.98), whereas the WLS association was significant (HR, 1.99; 95% CI, 1.37 to 2.88). In males with multimorbidity, the ALS in the least healthy group showed an HR of 1.71 (95% CI, 1.34 to 2.17), compared with a WLS HR of 1.61 (95% CI, 1.18 to 2.21).

### Years of life lost by lifestyle scores and morbidity status

**Figure 4.**
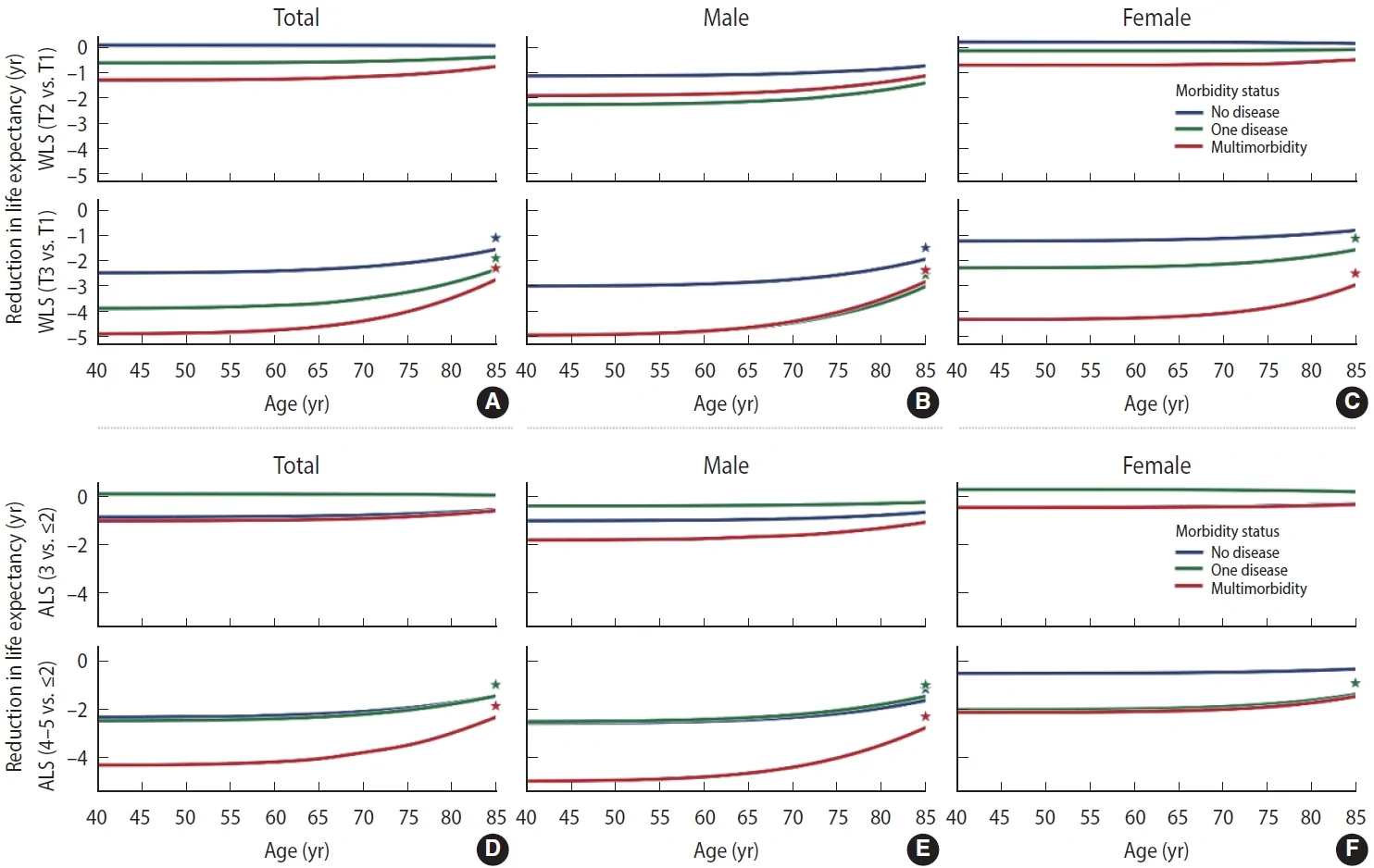
YLL by lifestyle categories and morbidity status. Tertiles of lifestyle quality were derived from the composite WLS, with T1 representing the healthiest lifestyle and T3 representing the least healthy lifestyle (A: total, B: male, C: female). The ALS ranges from 0 to 5 and was calculated by summing binary exposures (0/1) for the following behaviours: smoking, alcohol use (current drinking), poor diet quality (DQI-K ≤5), low physical activity (MET-hr/day <3), and short or long sleep duration (D: total, E: male and F: female). Participants received 1 point for each risk behaviour present. RYL estimates were obtained using multivariable-adjusted survival models that accounted for age, sex (in both-sex combined models), energy intake, BMI, WHR, education, income, marital status, and region of residence. YLL was computed as the difference in RYL between each lifestyle category and the healthiest reference group (WLS: T1; ALS: ≤2). WLS, weighted lifestyle score; ALS, accumulated lifestyle score; YLL, years of life lost; RYL, remaining years of life; T1, tertile 1 of the WLS; T2, tertile 2 of the WLS; T3, tertile 3 of the WLS. *p<0.05; all other values are presented as numbers.

To quantify the public health impact, we estimated YLL by comparing the least healthy WLS tertile (T3) with the healthiest tertile (T1) across ages 40 years to 85 years (Figure 4). Life expectancy differences remained stable between ages 40 years and 70 years before narrowing at older ages. At age 40 years, a higher WLS was associated with increasing YLL as morbidity burden worsened; losses ranged from 2.5 years among participants without chronic disease to 4.9 years among those with multimorbidity (Supplementary Material 6). The greatest magnitude of loss was observed in males with 1 chronic disease (5.0 years) and in males with multimorbidity (4.9 years). Although the ALS analysis showed a similar gradient, the magnitude of life-years lost was smaller across all groups (Supplementary Material 7).

Excluding deaths that occurred within the first 2 years of follow-up did not change the associations for either score (Supplementary Materials 8 and 9).

## DISCUSSION

We examined the association between lifestyle, measured using a WLS and an ALS, and mortality across morbidity strata in a Korean cohort. A less healthy lifestyle was associated with higher mortality in both males and females. The strongest associations were observed among participants with chronic disease, particularly those with multimorbidity. Among participants with multimorbidity, a less healthy lifestyle was associated with up to 5 years of reduced life expectancy before age 70 years.

In males, a less healthy lifestyle was associated with higher mortality across all morbidity groups, with stronger effects among those with chronic disease. This may reflect the high prevalence of smoking and heavy alcohol consumption among Korean males [24], which have been linked to vascular ageing through oxidative stress and inflammation [37]. These pathways may amplify the mortality effects of unhealthy behaviours across disease groups.

In the multimorbidity group, females showed a higher mortality risk than males. These findings should be interpreted with caution because the estimates in females suggest lower precision, possibly owing to the smaller sample size. Age-related factors, such as postmenopausal hormonal changes, could plausibly modify vulnerability to lifestyle-related risk [38]. However, these mechanisms remain speculative and require further investigation.

Similar sex-specific patterns were reported by Chudasama et al. [21], who found that lifestyle–mortality gradients were steeper in females with multimorbidity and flatter in males, supporting the view that biological and behavioural factors jointly modify the impact of lifestyle on survival. Our study extends this evidence by quantifying the same relationship in an East Asian population, and by incorporating sleep and a comprehensive dietary assessment, neither of which was included in previous weighted indices [21].

The ALS was included as a foundational benchmark to ensure that our results are comparable with the existing literature, which predominantly uses simple-count indices [20]. This approach allows our findings to be translated into the familiar framework of “behaviour counts” for public health messaging. However, the WLS provides additional value by accounting for the unequal prognostic contributions of individual behaviours, such as the greater impact of smoking relative to other factors [21]. By incorporating these relative biological weights, the WLS may improve risk discrimination and better reflect real-world patterns of mortality risk. Although cohort-specific weighting may limit direct generalisability to other populations, it ensures maximum relevance to Korean adults and provides a methodological template for future high-precision studies.

The life expectancy findings are consistent with the mortality results. Life expectancy gaps between healthy and unhealthy categories (WLS: T3 vs. T1; ALS: 4–5 vs. ≤2) showed minimal variation between ages 40 years and 70 years and then narrowed thereafter, especially in the multimorbidity group, indicating that lifestyle-related differences in longevity diminish at advanced ages. This convergence at older ages may reflect the reduced effect of the lifestyle–longevity association in later life [39], rather than a true disappearance of lifestyle impact. A Dutch population-based study similarly reported a 4–5-year life expectancy difference at age 45 years that declined to 2–3 years by age 85 years [40], consistent with our findings.

Previous cohort studies have shown that healthy lifestyles are associated with longer life expectancy [8,21]. In the United States Nurses’ Health Study and Health Professionals Follow-up Study, adherence to 5 low-risk lifestyle factors—healthy diet, regular exercise, non-smoking, moderate alcohol consumption, and healthy BMI—was associated with up to 14 additional years of life expectancy compared with adherence to none of these factors [41]. Asian cohorts, including the China Kadoorie Biobank and the Singapore Chinese Health Study, observed 6–9 additional years of life expectancy among participants who followed multiple healthy behaviours, including non-smoking, alcohol abstinence, physical activity, healthy eating, and maintenance of a healthy body shape, compared with participants who followed none [7,8]. Similar trends were observed in our study, despite differences in baseline age, inclusion of sleep in the lifestyle score, and use of morbidity-stratified analysis.

A recent UK Biobank study that applied a weighted lifestyle index among individuals with multimorbidity found that adults maintaining healthier lifestyles, including physical activity, non-smoking, a balanced diet, and moderate alcohol consumption, lived 6–7 years longer than those with similar chronic conditions but poorer lifestyle habits [21]. Although that analysis excluded sleep and dichotomised morbidity as no or 1 disease versus 2 or more diseases, the observed patterns were similar to those in our study. Application of a WLS may be particularly relevant in Korea, given its rapid demographic shift [19] and unique patterns of sex-specific lifestyle risk clustering [42,43]. In such contexts, weighted indices may provide a more precise assessment of lifestyle-related risk and help identify high-risk subgroups [21].

This study has several strengths. It is the first Korean cohort study to quantify life expectancy differences by lifestyle quality while applying a weighted index to improve precision over unweighted scores [21]. Furthermore, the morbidity-stratified analysis highlights that lifestyle modification remains a critical component of disease management, not only prevention.

Nonetheless, several limitations should be acknowledged. First, lifestyle and morbidity were assessed only at baseline, which introduces the possibility of misclassification. Evidence indicates that substantial proportions of adults alter their health behaviours over a decade [44]. This may lead to underestimation of the true association when only a single measurement is used, potentially biasing the results towards the null. Furthermore, although sensitivity analyses excluding deaths within 2 years yielded consistent results, reverse causation from long-term preclinical disease cannot be entirely ruled out [45,46]. Future studies using time-varying covariates that incorporate repeated measures of lifestyle and health status would be valuable for accounting for these changes over time. Second, as with all observational studies, residual confounding from unmeasured factors, such as psychosocial stress, depressive symptoms, and healthcare access, cannot be fully excluded despite covariate adjustment. Third, our assessment of multimorbidity relied on a simple sum of 18 self-reported and objectively measured chronic conditions. Although this disease-count approach is widely validated for its association with mortality [47], it may not differentiate between levels of disease severity or capture the synergistic effects of specific disease clusters. Treating all disease combinations as equivalent may mask the higher risks associated with more severe clinical profiles. This likely resulted in conservative estimation of the true mortality risk, particularly for individuals with high-severity clusters [48]. Future research should move beyond simple counts to incorporate severity-weighted indices, such as the Charlson comorbidity index, or use data-driven approaches, such as latent class analysis, to identify high-risk disease clusters. Fourth, because the cohort comprised middle-aged and older Korean adults, generalisability to younger or non-Asian populations may be limited.

In this Korean cohort, less healthy lifestyle patterns, as assessed using a weighted lifestyle score, were associated with higher all-cause mortality among adults with and without multimorbidity. The association was particularly pronounced among participants with multimorbidity. These findings suggest that lifestyle remains an important prognostic factor even in the presence of multiple chronic conditions and that public health strategies to promote healthier behaviours may help reduce mortality risk and extend life expectancy in ageing populations.

## Figures and Tables

**Figure f5-epih-48-e2026022:**
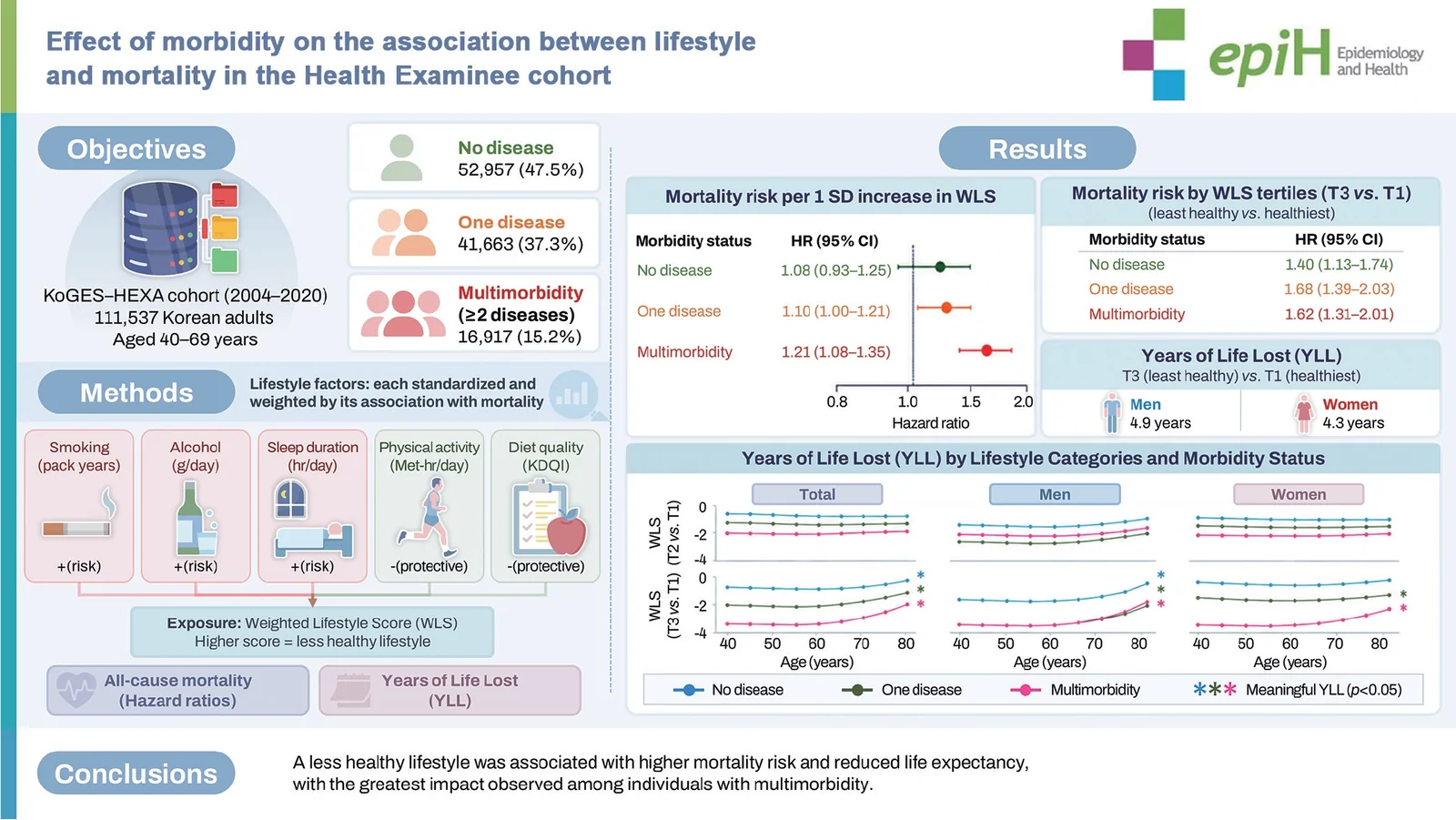


**Table 1.** Participant characteristics by WLS^1^ tertiles and sex

Characteristics	Male (n=37,842)			Female (n=73,695)		
	T1	T2	T3	T1	T2	T3
Demographics						
Participants (n)	3,147	4,652	30,043	34,031	32,529	7,135
Age (yr)	57.0 (51.0–63.0)	54.0 (47.0–62.0)	53.0 (46.0–60.0)	53.0 (48.0–59.0)	52.0 (46.0–58.0)	49.0 (44.0–54.0)
Events (n)	121	208	1677	627	576	157
Education status						
≤High school graduate	45.4	47.5	56.7	76.6	73.7	76.4
>High school graduate	54.6	52.5	43.3	23.4	26.3	23.6
Residence						
Urban	64.7	68.2	66.3	71.3	69.1	67.8
Rural	35.3	31.8	33.7	28.7	30.9	32.2
Marital status						
Married/Cohabiting	95.0	94.8	94.1	89.5	86.7	79.4
Single/Separated	5.0	5.2	5.9	10.5	13.3	20.6
Income (million KRW)						
<3	48.3	48.3	51.1	54.3	55.9	56.0
≥3	51.7	51.7	48.9	45.7	44.1	44.0
BMI						
Normal	30.2	31.7	27.9	43.4	43.0	43.5
Low	1.3	1.7	1.2	1.8	2.4	2.3
High	68.5	66.6	70.9	54.8	54.6	54.2
WHR^2^						
Normal	57.7	57.2	49.9	25.9	26.9	27.2
Above normal	42.3	42.8	50.1	74.1	73.1	72.8
Morbidity status						
None	37.3	41.0	37.4	50.9	53.2	56.4
One	42.3	41.8	44.8	34.3	33.5	33.2
Two or more	20.4	17.2	17.8	14.8	13.3	10.4
Total energy	1,770 (1,503–2,066)	1,724 (1,488–2,071)	1,795 (1,530–2,126)	1,640 (1,350–1,937)	1,635 (1,367–1,972)	1,699 (1,328–1,993)

Values are presented as percentage or median (interquartile range), unless otherwise indicated. WLS, weighted lifestyle score; KRW, Korean won; BMI, body mass index; WHR, waist–hip ratio.

1 WLS was calculated by standardising each lifestyle factor as a z-score and weighting each factor using standardised regression coefficients from cubic spline models adjusted for sex, BMI, WHR, energy intake, education, income, marital status, morbidity status, and region; Higher scores indicate less healthy lifestyles; WLS tertiles were defined as follows: T1=healthiest lifestyle; T3=least healthy lifestyle.

2 WHR categories were defined as normal (<90 cm for males, <80 cm for females) and above normal (≥90 cm for males, ≥80 cm for females).

## Data Availability

Data from the Korean Genome and Epidemiology Study (KoGES) are managed by the Clinical and Omics Data Archive (CODA) at the Korea Disease Control and Prevention Agency. Korean law restricts public release of individual-level health data. Data are accessible for analysis on the CODA virtual platform, and access requests can be submitted through CODA at https://coda.nih.go.kr/frt/index.do.

## Supplementary materials

Supplementary Material 1. Construction of the Weighted lifestyle score (WLS): weights, derivation process

Supplementary Material 2. Summarizing variable definitions

Supplementary Material 3. Chronic diseases distribution at baseline

Supplementary Material 4. HRs for mortality by weighted lifestyle score according to morbidity status

Supplementary Material 5. HRs for mortality by accumulated lifestyle score according to morbidity status

Supplementary Material 6. Years of life lost by lifestyle categories and morbidity status-WLS

Supplementary Material 7. Years of life lost by lifestyle categories and morbidity status-ALS

Supplementary Material 8. HRs for mortality by weighted lifestyle score according to morbidity status after excluding 2 years of follow-up

Supplementary Material 9. HRs for mortality by accumulated lifestyle score according to morbidity status after of first 2 years of follow-up
