# Supplementary material for: Effect of morbidity on the association between lifestyle and mortality in the Health Examinee cohort
Source: Epidemiol Health. 2026 May 27;48:e2026022. doi: 10.4178/epih.e2026022 (PMC13503064; doi:10.4178/epih.e2026022)
Supplement: Supplementary file 1 [file epih-48-e2026022-Supplementary-1.docx]

**Supplementary Material 1**. Construction of the Weighted lifestyle score (WLS): weights, derivation process
