# Supplementary material for: Effect of morbidity on the association between lifestyle and mortality in the Health Examinee cohort
Source: Epidemiol Health. 2026 May 27;48:e2026022. doi: 10.4178/epih.e2026022 (PMC13503064; doi:10.4178/epih.e2026022)
Supplement: Supplementary file 2 [file epih-48-e2026022-Supplementary-2.docx]

**Supplementary Material 2**. Summarizing variable definitions

| Factor | WLS (Continuous) | ALS* (Binary) | Rationale |
| --- | --- | --- | --- |
| Smoking | Pack-years | Never (0) vs Former/Current (1) | Strongest mortality driver |
| Alcohol | g/day | Never/past (0) vs Current (1) | Dose-response relationship |
| Physical activity | MET-h/day | ≥3 (0) vs <3 (1) | Guideline threshold |
| Sleep | Hours/night | 7-8h (0) vs <7/>8h (1) | U-shaped mortality risk |
| Diet | DQI-K (0-9) | >5 (0) vs ≤5 (1) | Validated Korean threshold |

*A score for each individual was created by summing the 1s and 0s for each lifestyle factor. The higher the lifestyle value, the unhealthier the lifestyle
