# Supplementary material for: Effect of morbidity on the association between lifestyle and mortality in the Health Examinee cohort
Source: Epidemiol Health. 2026 May 27;48:e2026022. doi: 10.4178/epih.e2026022 (PMC13503064; doi:10.4178/epih.e2026022)
Supplement: Supplementary file 3 [file epih-48-e2026022-Supplementary-3.docx]

**Supplementary Material 3.** Chronic diseases distribution at baseline

|  |  | One disease (41,663) | | |  | Multimorbidity (16,917) | | | |
| --- | --- | --- | --- | --- | --- | --- | --- | --- | --- |
| No. | **Condition** | **Total** | **Men** | **Women** |  | **Combinations** | **Total** | **Men** | **Women** |
| 1 | Hypertension | 76.4 | 80.9 | 74.4 |  | Hypertension + Diabetes | 20.4 | 27.8 | 15.5 |
| 2 | Diabetes | 5.3 | 6.4 | 4.5 |  | Hypertension + Hyperlipidemia | 9.8 | 7.5 | 11.4 |
| 3 | Hyperlipidemia | 2.1 | 1.3 | 2.6 |  | Hypertension + chronic kidney disease | 9.6 | 13.4 | 7.1 |
| 4 | Chronic Kidney Disease | 3.1 | 3.2 | 3.0 |  | Hypertension + Arthritis | 5.7 | 1.9 | 8.3 |
| 5 | Arthritis | 2.4 | 0.7 | 3.6 |  | Hypertension +Myocardial Infarctions | 3.1 | 4.1 | 2.4 |
| 6 | Osteoporosis | 1.6 | 0.1 | 2.5 |  | Hypertension + Diabetes + Hyperlipidemia | 3.0 | 2.8 | 3.1 |
| 7 | Thyroid Disease | 2.1 | 0.4 | 3.1 |  | Hypertension + Osteoporosis | 2.9 | 0.2 | 4.7 |
| 8 | Myocardial Infarctions | 0.9 | 1.4 | 0.8 |  | Hypertension + Thyroid disease | 2.8 | 0.8 | 4.1 |
| 9 | Chronic Gastritis | 1.2 | 0.8 | 1.6 |  | Hypertension + Diabetes + chronic kidney disease | 2.0 | 2.8 | 1.5 |
| 10 | Cancer | 1.3 | 0.6 | 1.7 |  | Hypertension + Gastritis | 1.8 | 1.5 | 2.0 |
| 11 | Glaucoma | 0.4 | 0.5 | 0.6 |  | Hypertension + Cancer | 1.8 | 1.1 | 2.2 |
| 12 | Prostate Hypertrophy | 1.9 | 2.0 | 0.0 |  | Hypertension + Prostate Hypertrophy | 1.4 | 3.6 | 0.0 |
| 13 | Stroke | 0.2 | 0.3 | 0.1 |  | Hypertension + Glaucoma | 1.4 | 1.5 | 1.3 |
| 14 | Ulcers | 0.3 | 0.4 | 0.3 |  | Hypertension + Stroke | 1.2 | 1.7 | 0.8 |
| 15 | Asthma | 0.2 | 0.3 | 0.4 |  | Diabetes + Hyperlipidemia | 1.2 | 1.2 | 1.1 |
| 16 | Gout | 0.1 | 0.2 | 0.0 |  | Hypertension + Diabetes + Myocardial Infarctions | 0.9 | 1.3 | 0.5 |
| 17 | Bronchitis | 0.1 | 0.1 | 0.1 |  | Hypertension + Asthma | 0.8 | 0.6 | 1.0 |
| 18 | Gall Stones | 0.1 | 0.1 | 0.1 |  | Hypertension + Hyperlipidemia + chronic kidney disease | 0.8 | 0.9 | 0.7 |
| 19 | Cystitis | 0.1 | 0.1 | 0.3 |  | Diabetes + chronic kidney disease | 0.7 | 1.1 | 0.5 |
| 20 | COPD | 0.1 | 0.1 | 0.2 |  | Arthritis + Osteoporosis | 0.7 | 0.0 | 1.2 |
| 21 | Parkinson's | 0.1 | 0.1 | 0.1 |  | Hypertension + Hyperlipidemia + Myocardial Infections | 0.7 | 0.8 | 0.7 |
| 22 | Fatty Liver | 0.0 | 0.0 | 0.0 |  | Others* | 27.3 | 23.4 | 29.9 |

* Includes all remaining multimorbidity combinations beyond those listed, comprising participants with 2–9 coexisting chronic diseases. The distribution of these combinations was as follows:

Overall, 2 diseases (12,625; 20.37%), 3 diseases (3,280; 5.29%), 4 diseases (784; 1.27%), 5 diseases (164; 0.26%), 6 diseases (30; 0.05%), 7 diseases (8; 0.01%), 8 diseases (2; 0.003%), and 9 diseases (1; 0.002%).

By sex:

Men had 2 diseases (5,152; 17.81%), 3 diseases (1,242; 4.29%), 4 diseases (292; 1.01%), 5 diseases (63; 0.22%), 6 diseases (14; 0.05%), 7 diseases (5; 0.02%), and 9 diseases (1; 0.003%);

Women had 2 diseases (7,456; 22.01%), 3 diseases (2,034; 6.01%), 4 diseases (494; 1.46%), 5 diseases (101; 0.30%), 6 diseases (16; 0.05%), 7 diseases (3; 0.01%), and 8 diseases (2; 0.01%). Percentages represent proportions within the multimorbidity group (n = 16,917).
