# Supplementary material for: Effect of morbidity on the association between lifestyle and mortality in the Health Examinee cohort
Source: Epidemiol Health. 2026 May 27;48:e2026022. doi: 10.4178/epih.e2026022 (PMC13503064; doi:10.4178/epih.e2026022)
Supplement: Supplementary file 4 [file epih-48-e2026022-Supplementary-4.docx]

**Supplementary Material 4. HRs for mortality by weighted lifestyle score according to morbidity status**

|  | **Total** | | | |  | **Men** | | | |  | **Women** | | | |
| --- | --- | --- | --- | --- | --- | --- | --- | --- | --- | --- | --- | --- | --- | --- |
|  | **Person years** | **Events (n)** | **HR (95% CI)** | **P trend^1^** |  | **Person years** | **Events (n)** | **HR (95% CI)** | **P trend^1^** |  | **Person years** | **Events (n)** | **HR (95% CI)** | **P trend^1^** |
| No disease | **Total (n=52,957)** | | | |  | **Men (n=14,325)** | | | |  | **Women (n= 38,632)** | | | |
| Continuous (per 1-SD increment) | 565,262.5 | **977** | 1.08 (0.93, 1.25) |  |  | 152,300.8 | 522 | **1.14 (1.03, 1.25)** |  |  | 412,961.7 | 455 | 1.10 (0.80, 1.38) |  |
| Tertile 1 (Healthy) | 199,549.3 | 245 | 1.00 (ref) | 0.03 |  | 12,656.8 | 32 | 1.00 (ref) | 0.01 |  | 186,892.5 | 213 | 1.00 (ref) | 0.15 |
| Tertile 2 | 203,235.9 | 241 | 0.92 (0.76, 1.11) |  |  | 20,409.3 | 55 | 1.08 (0.69, 1.70) |  |  | 182,826.6 | 186 | 0.88 (0.72, 1.09) |  |
| Tertile 3 (Unhealthy) | 162,477.3 | 491 | **1.40 (1.13, 1.74)** |  |  | 119,234.7 | 435 | **1.52 (1.06, 2.18)** |  |  | 43,242.6 | 56 | **1.39 (1.01, 1.92)** |  |
| One disease | **Total (n=41,663)** | | | |  | **Men (n=16,727)** | | | |  | **Women (n=24,936)** | | | |
| Continuous (per 1-SD increment) | 441,718.4 | 1,350 | 1.10 (1.00, 1.21) |  |  | 176,463.7 | 825 | **1.12 (1.05, 1.20)** |  |  | 265,254.7 | 525 | 1.05 (0.80, 1.38) |  |
| Tertile 1 (Healthy) | 139,655.1 | 287 | 1.00 (ref) | <0.01 |  | 14,259.1 | 44 | 1.00 (ref) | <0.01 |  | 125,396.0 | 243 | 1.00 (ref) | <0.01 |
| Tertile 2 | 135,309.9 | 307 | 1.11 (0.94, 1.32) |  |  | 20,914.8 | 85 | 1.47 (1.00, 2.16) |  |  | 114,395.1 | 222 | 1.04 (0.85, 1.26) |  |
| Tertile 3 (Unhealthy) | 166,753.4 | 756 | **1.68 (1.39, 2.03)** |  |  | 141,289.8 | 696 | **1.95 (1.41, 2.70)** |  |  | 25,463.6 | 60 | **1.73 (1.28, 2.33)** |  |
| Multimorbidity | **Total (n=16,917)** | | |  |  | **Men (n=6,790)** | | | |  | **Women (n=10,127)** | | | |
| Continuous (per 1-SD increment) | 174,215.2 | 1,039 | **1.21 (1.08, 1.35)** |  |  | 69,142.5 | 659 | **1.14 (1.06, 1.22)** |  |  | 105,072.7 | 380 | **1.73 (1.13, 2.64)** |  |
| Tertile 1 (Healthy) | 59,450.1 | 216 | 1.00 (ref) | <0.01 |  | 6,657.1 | 45 | 1.00 (ref) | 0.01 |  | 52,793.0 | 171 | 1.00 (ref) | <0.01 |
| Tertile 2 | 52,816.9 | 236 | 1.17 (0.97, 1.43) |  |  | 8,186.5 | 68 | 1.27 (0.84, 1.88) |  |  | 44,630.4 | 168 | 1.15 (0.92, 1.45) |  |
| Tertile 3 (Unhealthy) | 61,948.2 | 587 | **1.62 (1.31, 2.01)** |  |  | 54,298.9 | 546 | **1.61 (1.18, 2.21)** |  |  | 7,649.3 | 41 | **1.99 (1.37, 2.88)** |  |

HR, hazard ratio; CI, confidence interval; SD, standard deviation; WLS, weighted lifestyle score; BMI, body mass index; WHR, waist–hip ratio. The WLS was developed to assess the varying impact of lifestyle factors on mortality risk. Each lifestyle factor was standardised as a z-score and weighted using regression coefficients derived from cubic spline models adjusted for sex, BMI, WHR, energy intake, morbidity status, education, income, marital status, and region. The weighted scores were then summed to calculate the WLS. HRs and 95% CIs were adjusted for energy intake, sex (in both-sex combined models), education, region of residence, marital status, income, BMI, morbidity status, and WHR. Tertile 1 indicates the healthiest lifestyle, and tertile 3 indicates the least healthy lifestyle.. ^1^p-values were derived from models treating WLS as an ordinal variable to assess linear trends across tertiles.
