# Supplementary material for: Effect of morbidity on the association between lifestyle and mortality in the Health Examinee cohort
Source: Epidemiol Health. 2026 May 27;48:e2026022. doi: 10.4178/epih.e2026022 (PMC13503064; doi:10.4178/epih.e2026022)
Supplement: Supplementary file 5 [file epih-48-e2026022-Supplementary-5.docx]

**Supplementary Material 5. HRs for mortality by accumulated lifestyle score according to morbidity status**

|  | Total | | | |  | Men | | | |  | Women | | | |
| --- | --- | --- | --- | --- | --- | --- | --- | --- | --- | --- | --- | --- | --- | --- |
|  | **Person years** | **Events (n)** | **HR^a^ (95% CI)** | **P trend^1^** |  | **Person years** | **Events (n)** | **HR^a^ (95% CI)** | **P trend^1^** |  | **Person years** | **Events (n)** | **HR^a^ (95% CI)** | **P trend^1^** |
| No disease | **Total (n=52,957)** | | | |  | **Men (n=14,325)** | | | |  | **Women (n= 38,632)** | | | |
| ≤2 (Healthy) | 253,072.50 | 316 | 1. 00 (ref) |  |  | 24,415.90 | 65 | 1. 00 (ref) |  |  | 228,656.60 | 251 | 1. 00 (ref) |  |
| 3 | 186,310.50 | 297 | 1.14 (0.97, 1.34) | <0.01 |  | 45,942.00 | 139 | 1.19 (0.89, 1.60) | 0.01 |  | 140,368.50 | 158 | 1.13 (0.93, 1.39) | 0.24 |
| 4-5 (Unhealthy) | 125,879.50 | 364 | **1.41 (1.18, 1.69)** |  |  | 81,942.90 | 318 | **1.54 (1.17, 2.01)** |  |  | 43,936.60 | 46 | 1.17 (0.85, 1.60) |  |
| One disease | **Total (n=41,663)** | | | |  | **Men (n=16,727)** | | | |  | **Women (n=24,936)** | | | |
| ≤2 (Healthy) | 178,018.90 | 420 | 1. 00 (ref) |  |  | 25,305.00 | 110 | 1. 00 (ref) |  |  | 152,713.90 | 310 | 1. 00 (ref) |  |
| 3 | 141,439.10 | 376 | 0.98 (0.85, 1.13) | 0.02 |  | 54,226.30 | 221 | 1.04 (0.82, 1.30) | 0.04 |  | 87,212.80 | 155 | 0.93 (0.77, 1.13) | 0.05 |
| 4-5 (Unhealthy) | 122,260.40 | 554 | **1.33 (1.14, 1.55)** |  |  | 96,932.40 | 494 | **1.34 (1.09, 1.66)** |  |  | 25,328.00 | 60 | **1.44 (1.08, 1.90)** |  |
| Multimorbidity | **Total (n=16,917)** | | | |  | **Men (n=6,790)** | | | |  | **Women (n=10,127)** | | | |
| ≤2 (Healthy) | 73,948.40 | 302 | 1. 00 (ref) |  |  | 11,254.70 | 81 | 1. 00 (ref) |  |  | 62,693.70 | 221 | 1. 00 (ref) |  |
| 3 | 55,213.70 | 295 | 1.12 (0.95, 1.32) | <0.01 |  | 20,788.90 | 167 | 1.23 (0.94, 1.60) | <0.01 |  | 34,424.80 | 128 | 1.08 (0.87, 1.35) | 0.11 |
| 4-5 (Unhealthy) | 45,053.10 | 442 | **1.57 (1.32, 1.87)** |  |  | 37,098.90 | 411 | **1.71 (1.34, 2.17)** |  |  | 7,954.20 | 31 | 1.34 (0.93, 1.98) |  |

HR, hazard ratio; ALS, accumulated lifestyle score; BMI, body mass index; WHR, waist–hip ratio. 1p-values represent the p for linear trend across increasing numbers of unhealthy lifestyle factors, grouped as ≤2, 3, and 4–5.

HRs were adjusted for energy intake, sex (in both-sex combined models), education, region of residence, marital status, income level, BMI, and WHR. The ALS ranges from 0 to 5 and was calculated by summing binary exposures (0/1) for the following behaviours: smoking, alcohol use (current drinking), poor diet quality (DQI-K ≤5), low physical activity (MET-hr/day <3), and short or long sleep duration. Participants received 1 point for each risk behaviour present.

^1^p-values represent the p for linear trend across increasing numbers of unhealthy lifestyle factors, grouped as ≤2, 3, and 4–5.
