# Supplementary material for: Effect of morbidity on the association between lifestyle and mortality in the Health Examinee cohort
Source: Epidemiol Health. 2026 May 27;48:e2026022. doi: 10.4178/epih.e2026022 (PMC13503064; doi:10.4178/epih.e2026022)
Supplement: Supplementary file 6 [file epih-48-e2026022-Supplementary-6.docx]

**Supplementary Material 6**. Years of life lost by lifestyle categories and morbidity status-WLS

|  | Total At 40 years | |  | Men At 40 years | |  | Women At 40 years | |
| --- | --- | --- | --- | --- | --- | --- | --- | --- |
|  | RYL | YLL (Years, 95% CI) |  | RYL | YLL (Years, 95% CI) |  | RYL | YLL (Years, 95% CI) |
| No disease | | | | | | | | |
| Tertile 1 (Healthy) | 53.6 | Ref |  | 54.9 | Ref |  | 56.7 | Ref |
| Tertile 2 | 53.7 | 0.1 (-0.9, 1.0) |  | 53.8 | -1.2 (-3.3, 1.0) |  | 56.8 | 0.2 (-0.8, 0.4) |
| Tertile3 (Unhealthy) | 51.1 | **-2.5 (-3.7, -1.3)*** |  | 51.9 | **-3.0 (-4.8, -1.2)*** |  | 55.4 | -1.2 (-2.5, 0.0) |
| One disease | | | | | | | | |
| Tertile 1 (Healthy) | 51.5 | Ref |  | 52.9 | Ref |  | 54.9 | Ref |
| Tertile 2 | 50.8 | -0.7 (-1.8, 0.5) |  | 50.6 | -2.3 (-4.7, 0.1) |  | 54.7 | -0.2 (-1.0, 0.7) |
| Tertile3 (Unhealthy) | 47.6 | **-3.9 (-5.2, -2.6)*** |  | 47.9 | **-5.0 (-7.0, -3.0)*** |  | 52.6 | **-2.3 (-4.1, -0.5)*** |
| Multimorbidity | | | | | | | | |
| Tertile 1 (Healthy) | 48.6 | Ref |  | 49.8 | Ref |  | 53.1 | Ref |
| Tertile 2 | 47.2 | -1.3 (-2.9, 0.3) |  | 47.9 | -1.9 (-5.1, 1.2) |  | 52.4 | -0.7 (-2.0, 0.5) |
| Tertile3 (Unhealthy) | 43.7 | **-4.9 (-6.7, -3.1)*** |  | 44.8 | **-4.9 (-7.5, -2.4)*** |  | 48.8 | **-4.3 (-6.9, -1.7)*** |

WLS, Weighted Lifestyle Score; RYL, Remaining Years of Life; YLL, Years of Life Lost (difference in RYL compared to Tertile 1 Tertiles of lifestyle quality were derived from a composite lifestyle score)

Tertiles of lifestyle quality were derived from the composite WLS, with T1 representing the healthiest lifestyle and T3 representing the least healthy lifestyle.

RYL estimates were obtained using multivariable-adjusted survival models that accounted for age, sex (in both-sex combined models), energy intake, BMI, WHR, education, income, marital status, and region of residence.

YLL was computed as the difference in RYL between each lifestyle category and the healthiest reference group (WLS: T1)

^*^Significant years of life lost.
