# Supplementary material for: Effect of morbidity on the association between lifestyle and mortality in the Health Examinee cohort
Source: Epidemiol Health. 2026 May 27;48:e2026022. doi: 10.4178/epih.e2026022 (PMC13503064; doi:10.4178/epih.e2026022)
Supplement: Supplementary file 7 [file epih-48-e2026022-Supplementary-7.docx]

**Supplementary Material 7. Years of life lost by lifestyle categories and morbidity status-ALS**

|  | Total At 40 years | |  | Men At 40 years | |  | Women At 40 years | |
| --- | --- | --- | --- | --- | --- | --- | --- | --- |
| Lifestyle factor | **RYL** | **YLL (Years, 95% CI)** |  | **RYL** | **YLL (Years, 95% CI)** |  | **RYL** | **YLL (Years, 95% CI)** |
| No disease |  |  |  |  |  |  |  |  |
| ≤2 (Healthy) | 53.1 | 0.0 (ref) |  | 54.1 | 0.0 (ref) |  | 56.8 | 0.0 (ref) |
| 3 | 52.3 | -0.8 (-1.8, 0.2) |  | 53.1 | -1.0 (-2.6, 0.6) |  | 56.4 | -0.4 (-1.1, 0.3) |
| 4-5 (Unhealthy) | 50.8 | **-2.3 (-3.5, -1.2)^*^** |  | 51.5 | **-2.6 (-4.2, -1.0)^*^** |  | 56.3 | -0.5 (-1.6, 0.6) |
| One disease |  |  |  |  |  |  |  |  |
| ≤2 (Healthy) | 49.8 | 0.0 (ref) |  | 50.2 | 0.0 (ref) |  | 54.7 | 0.0 (ref) |
| 3 | 49.9 | 0.1 (-1.0, 1.3) |  | 49.9 | -0.3 (-2.2, 1.4) |  | 55.0 | 0.3 (-0.6, 1.2) |
| 4-5 (Unhealthy) | 47.3 | **-2.5 (-3.8, -1.2)^*^** |  | 47.7 | **-2.5 (-4.2, -0.8)^*^** |  | 52.7 | **-2.0 (-3.8, -0.3)^*^** |
| Multimorbidity |  |  |  |  |  |  |  |  |
| ≤2 (Healthy) | 47.3 | 0.0 (ref) |  | 49.1 | 0.0 (ref) |  | 52.7 | 0.0 (ref) |
| 3 | 46.3 | -1.0 (-2.5, 0.5) |  | 47.3 | -1.8 (-4.1, 0.5) |  | 52.3 | -0.4 (-1.8, 0.9) |
| 4-5 (Unhealthy) | 43.0 | **-4.3 (-6.0, -2.7)^*^** |  | 44.0 | **-5.1 (-7.1, -2.9)^*^** |  | 50.5 | -2.2 (-4.8, 0.5) |

ALS, Accumulated Lifestyle Score; RYL, Remaining Years of Life; YLL, Years of Life Lost (difference in RYL compared to the reference group);

The ALS ranges from 0 to 5 and was calculated by summing binary exposures (0/1) for the following behaviours: smoking, alcohol use (current drinking), poor diet quality (DQI-K ≤5), low physical activity (MET-hours/day <3), and short or long sleep duration. Participants received 1 point for each risk behaviour present. RYL estimates were obtained using multivariable-adjusted survival models that accounted for age, sex (in both-sex combined models), energy intake, BMI, WHR, education, income, marital status, and region of residence. YLL was computed as the difference in RYL between each lifestyle category and the healthiest reference group ( ALS ≤2).

^*^Significant years of life lost.
