# Supplementary material for: Effect of morbidity on the association between lifestyle and mortality in the Health Examinee cohort
Source: Epidemiol Health. 2026 May 27;48:e2026022. doi: 10.4178/epih.e2026022 (PMC13503064; doi:10.4178/epih.e2026022)
Supplement: Supplementary file 9 [file epih-48-e2026022-Supplementary-9.docx]

**Supplementary Material 9**. HRs for mortality by accumulated lifestyle score according to morbidity status after of first 2 years of follow-up

|  | Total | | | |  | Men | | | |  | Women | | | |
| --- | --- | --- | --- | --- | --- | --- | --- | --- | --- | --- | --- | --- | --- | --- |
|  | **Person years** | **Events (n)** | **HR^a^ (95% CI)** | **P trend^1^** |  | **Person years** | **Events (n)** | **HR^a^ (95% CI)** | **P trend^1^** |  | **Person years** | **Events (n)** | **HR^a^ (95% CI)** | **P trend^1^** |
| No disease | **Total (n=52,855)** | | | |  | **Men (n=14,268)** | | | |  | **Women (38,587)** | | | |
| ≤2 (Healthy) | 256,548.0 | 287 | 1. 00 (ref) | <0.01 |  | 24,409.3 | 59 | 1. 00 (ref) | 0.03 |  | 232,138.7 | 228 | 1. 00 (ref) | 0.17 |
| 3 | 186,268.8 | 259 | 1.10 (0.93, 1.31) |  |  | 45,921.0 | 121 | 1.15 (0.84, 1.57) |  |  | 140,347.8 | 138 | 1.09 (0.88, 1.35) |  |
| 4-5 (Unhealthy) | 125,838.6 | 329 | **1.43 (1.18, 1.72)** |  |  | 81,904.0 | 285 | **1.52 (1.15, 2.02)** |  |  | 43,934.6 | 44 | 1.24 (0.89, 1.72) |  |
| One disease | **Total (n=41,544)** | | | |  | **Men (n=16,646)** | | | |  | **Women (n=24,898)** | | | |
| ≤2 (Healthy) | 177,981.6 | 390 | 1. 00 (ref) |  |  | 25,288.2 | 96 | 1. 00 (ref) | 0.04 |  | 152,693.4 | 294 | 1. 00 (ref) | 0.25 |
| 3 | 141,396.3 | 341 | 0.97 (0.83, 1.12) | 0.04 |  | 54,201.5 | 201 | 1.09 (0.85, 1.39) |  |  | 87,194.8 | 140 | 0.89 (0.73, 1.09) |  |
| 4-5 (Unhealthy) | 122,196.0 | 500 | **1.32 (1.13, 1.55)** |  |  | 96,876.2 | 447 | **1.41 (1.13, 1.76)** |  |  | 25,319.8 | 53 | **1.35 (1.01, 1.82)** |  |
| Multimorbidity | **Total (n=16,824)** | | | |  | **Men (n=6,725)** | | | |  | **Women (n=10,099)** | | | |
| ≤2 (Healthy) | 73,921.7 | 281 | 1. 00 (ref) | <0.01 |  | 11,247.2 | 76 | 1. 00 (ref) | 0.01 |  | 62,674.5 | 205 | 1. 00 (ref) | 0.13 |
| 3 | 55,177.4 | 267 | 1.10 (0.92, 1.30) |  |  | 20,766.2 | 149 | 1.17 (0.89, 1.55) |  |  | 34,411.2 | 118 | 1.07 (0.85, 1.34) |  |
| 4-5 (Unhealthy) | 45,002.5 | 398 | **1.54 (1.28, 1.85)** |  |  | 37,051.1 | 369 | **1.64 (1.28, 2.10)** |  |  | 7,951.4 | 29 | 1.36 (0.92, 2.01) |  |

HR, hazard ratio; ALS, accumulated lifestyle score; BMI, body mass index; WHR, waist–hip ratio. 1p-values represent the p for linear trend across increasing numbers of unhealthy lifestyle factors, grouped as ≤2, 3, and 4–5.

HRs were adjusted for energy intake, sex (in both-sex combined models), education, region of residence, marital status, income level, BMI, and WHR. The ALS ranges from 0 to 5 and was calculated by summing binary exposures (0/1) for the following behaviours: smoking, alcohol use (current drinking), poor diet quality (DQI-K ≤5), low physical activity (MET-hr/day <3), and short or long sleep duration. Participants received 1 point for each risk behaviour present.

^1^p-values represent the p for linear trend across increasing numbers of unhealthy lifestyle factors, grouped as ≤2, 3, and 4–5.
